# Coordinated regulation of tomato sugar accumulation by relative humidity and field capacity through source–sink–transport balance

**DOI:** 10.3389/fpls.2026.1775354

**Published:** 2026-02-25

**Authors:** Wei Han, Xindi Zhang, Yinghui Hao, Chenxi Sun, Xiaoxin Lin

**Affiliations:** 1 Jiangsu Provincial University Key Laboratory of Agricultural and Ecological Meteorology, School of Ecology and Applied Meteorology, Nanjing University of Information Science & Technology, Nanjing, China; 2 Shandong Provincial Key Laboratory of Test Technology on Food Quality and Safety, Institute of Agricultural Product Quality and Standards, Shandong Academy of Agricultural Sciences, Jinan, China

**Keywords:** phloem transport, photosynthesis, sugar metabolic enzymes, tomato, transpiration, xylem flow

## Abstract

To clarify the regulatory mechanism underlying the interaction between air relative humidity (RH) and soil moisture (field capacity, FC) on sugar accumulation in tomato fruits, this study used two tomato cultivars, ‘Xiuzhen’ and ‘Jinhong 208’, as materials. Four RH levels (40%, 60%, 80%, and 95%) and two FC levels (40% and 80% FC) were established in artificial climate chambers to systematically analyze the relationships among photosynthesis, transpiration, vascular transport, sugar metabolism enzyme activity, and sugar accumulation. The results showed that low soil moisture (40% FC) reduced the photosynthetic rate but significantly increased sugar metabolism enzyme activity, thereby promoting sugar accumulation. In contrast, high soil moisture (80% FC) was associated with higher photosynthetic rates but lower sugar-metabolizing enzyme activity. High air humidity (95% RH) markedly associated with lower photosynthesis, stomatal conductance, and sugar metabolism enzyme activity, accompanied by a significant decrease (*P* < 0.01) in glucose, fructose, and sucrose contents; conversely, low humidity promoted sugar accumulation. Both ‘Xiuzhen’ and ‘Jinhong 208’ achieved the highest soluble sugar content under 40% RH and 40% FC conditions. Gray relational analysis revealed that FC had a stronger influence on photosynthesis, while RH had a more pronounced effect on sugar metabolism. Under sufficient water supply, air humidity management played a greater role in regulating fruit quality. Path analysis indicated that RH exerted significant negative effects on sugar accumulation via reduced transpiration (path coefficient = –0.590), sugar metabolism enzyme activity (–0.358), and phloem transport (–0.424). In contrast, FC promoted phloem transport (0.680) but somewhat associated with lower enzyme activity (–0.500). Humidity is closely related to sugar accumulation, but weakly related to yield and its components. This study reveals the specific strategies of sugar regulation among different tomato cultivars and provides insights for humidity and irrigation optimization in greenhouse tomato production.

## 1 Introduction

Tomatoes are valued for their nutritional content and characteristic flavor (Pott et al., 2019). As consumer demand for tomato quality increases, understanding the mechanisms of sugar accumulation in tomatoes becomes even more important. These sugars not only influence the taste and nutritional quality of tomatoes but also act as signaling molecules during development and ripening (Qin et al., 2016).

Relative air humidity (RH) and field capacity (FC) are widely recognized as two of the most critical environmental factors influencing water movement, photosynthesis, and water use efficiency (Du et al., 2018; Bwire et al., 2024). RH mainly reflects atmospheric evaporative demand, whereas FC represents soil water supply; their interaction shapes plant water status. Calvo-Polanco et al. (2017) demonstrated that air humidity has a significant impact on water transport within plants. Cui et al. (2020) found a positive correlation between field capacity and tomato yield, but a negative correlation between field capacity and both total soluble solids and soluble sugar content in tomatoes. While the individual effects of RH and FC have been extensively studied, their combined influence on sugar accumulation remains poorly understood. According to the source–sink theory, sugar transport in plants is governed by the movement and distribution of carbohydrates, produced via photosynthesis, to non-photosynthetic organs (Li et al., 2022). However, the mechanism by which RH and FC interact to regulate sugar metabolism is still not fully understood (Vories and Sudduth, 2021; Zheng Y. et al., 2022).

Therefore, based on the source–sink regulation theory, we hypothesized that relative humidity and soil moisture jointly influence sugar accumulation by regulating source activity (photosynthesis), sink strength (enzyme activity), and transport capacity (vascular flow) (Luo et al., 2021). The objectives of this study were: (1) Determine the effects of different combinations of RH and FC on sugar-related physiological processes during tomato fruit development. (2) Elucidate the relationships among sugar content, key enzymes involved in sugar metabolism, photosynthetic performance, and vascular transport, and quantify the relative contributions of these factors to sugar accumulation.

## 2 Materials and methods

### 2.1 Plant materials and treatments

This study was conducted using two tomato cultivars, ‘Xiuzhen’ and ‘Jinhong 208’, in a Venlo-type glass greenhouse, oriented north–south (30.0 m × 9.6 m × 5.0 m ridge height) at Nanjing University of Information Science & Technology from July 2023 to February 2024. Prior to the experiment, tomato seedlings were transplanted into plastic pots (height: 18.8 cm; base diameter: 14.5 cm). The growing medium consisted of garden soil mixed with a 2:1 substrate-to-soil ratio, with each pot containing 2.5 kg of loam soil. At the flowering and fruit-setting stage (55–68 days after transplanting, when the first flower cluster had set fruit), plants with uniform growth were selected and transferred to an artificial climate chamber (A1000 PG KIT, Canada) for environmental treatments. Four relative humidity (RH) levels (40% ± 5%, 60% ± 5%, 80% ± 5%, and 95% ± 5%) were selected to represent the range commonly observed in greenhouse production (Lind et al., 2016; Stuerz and Asch, 2019). Soil moisture was maintained at two levels, 40% ± 5% and 80% ± 5% of field capacity (FC), to simulate moderate water deficit and well-irrigated conditions, respectively (Yuan et al., 2016). These two FC levels were selected to represent contrasting water-deficit and well-watered conditions and to facilitate the detection of RH × FC interaction effects under controlled conditions. The combination of these factors resulted in eight treatments, allowing the evaluation of both individual and interactive effects of atmospheric and soil water conditions on tomato performance. Each treatment was replicated three times. The climate chamber was programmed with a 12-hour photoperiod (08:00–20:00) and a photosynthetically active radiation (PAR) level of 1000 μmol m^−^² s^−^¹. The day/night temperature was set at 25 °C/15 °C. To ensure the accuracy of the soil moisture measurements, the time-domain reflectometer was periodically calibrated during the experiment using the drying method (An et al., 2025). For each measurement, three fruits were randomly selected from the experimental plants, and the sampling time was randomly determined to minimize sampling bias. Although relative humidity and soil moisture fluctuate diurnally in greenhouses, fixed RH and field capacity levels were used to isolate their effects on tomato sugar accumulation and serve as a reference for future dynamic studies. The 95% RH treatment represents an extreme but realistic high-humidity condition.

### 2.2 Gas exchange parameters

Physiological measurements were conducted on the central part of the fifth fully expanded leaf and the equatorial region of mature fruits. Net photosynthetic rate (Pn), stomatal conductance (Gs), intercellular CO_2_ concentration (Ci), and transpiration rate (Tr) were measured (Zhang et al., 2021) using a portable photosynthesis system (LI-6400, LI-COR Biosciences, Lincoln, NE, USA). Measurements were performed at approximately 10:00 a.m. During measurements, leaves were exposed to a photosynthetic photon flux density (PPFD) of 1000 μmol·m⁻²·s⁻¹ and a CO_2_ concentration of 380 ± 10 μmol·mol^-1^.

### 2.3 SPAD values

Chlorophyll meter (SPAD) values were assessed using a SPAD-502 meter (Konica Minolta, Japan) (Zając and Skrajna, 2024). All measurements were conducted between 09:00 and 13:00 under stable light and temperature conditions. To ensure data accuracy and reproducibility, each parameter was measured with three biological replicates.

### 2.4 Fruit transpiration

Measurements were conducted using an open differential system, based on the method of Morales et al. (2022) with slight modifications. At the maturation stage (when the fruit surface is fully red), fruits of similar size and color were selected (Su et al., 2022). Before 09:00 each day, the measurement device was attached to the target fruits and left in place for 1.5 hours before data collection. The measurement chamber was constructed from a 15 × 15 × 15 cm acrylic (methacrylate) box. The air inside the chamber was pre-conditioned to 40% RH using a condensation trap and supplied at a flow rate of 1 L·min^−^¹. The relative humidity (RH) of the reference air and air leaving the chamber was monitored using a TH20BL-EX-H humidity sensor (Huahanwei, China). For a comprehensive and detailed description of the measurement apparatus, please refer to Morales et al. (2022). The fruit transpiration rate was calculated based on the relative humidity difference.

### 2.5 Measurement of flow rates

The method was based on Winkler and Knoche (2021) and Giovannini et al. (2022) with slight modifications. At the fruit maturation stage, the phloem outflow, xylem inflow/outflow, and transpiration outflow at the fruit pedicel were measured. Fruits of uniform size and color were selected from each plant and subjected to the following treatments: intact, no intervention, maintaining normal vascular connections; girdled, the phloem connection at the pedicel was severed; severed, all vascular connections at the pedicel were cut, and the cut surface was coated with glue and reattached to its original position. Each treatment lasted 3 days, during which the daily variation in fruit diameter was measured and converted to fruit weight using the following formula to calculate the 24-hour phloem and xylem flow rates, expressed in mg·g^−^¹·min^−^¹:


$$W\left(g\right)=a\times D{\left(mm\right)}^{b}$$

Where: W = fruit weight (g); D = fruit diameter (mm); a and b are constants, with values of 0.0023 (SE ± 0.00025) and 2.7734 (SE ± 0.032), respectively.

### 2.6 Determination of soluble sugars, their components, and yield

Soluble sugar content was determined using the anthrone colorimetric method, as described by Yang et al. (2023) and Cole et al. (1954). A 0.05 g portion of dried fruit sample was ground and transferred into a centrifuge tube. Then, 5–6 mL of distilled water was added, and the tube was placed in a boiling water bath at 100 °C for 30 minutes. After heating, the sample was centrifuged at 4000 rpm for 10 minutes. The supernatant was transferred to a 25 mL volumetric flask and brought to volume with distilled water. A 0.1 mL aliquot of the extract was then mixed with 3.0 mL of anthrone reagent in a glass test tube and incubated in a 90 °C water bath for 30 minutes. Absorbance was measured at 620 nm, and soluble sugar concentration was calculated using a standard curve. Results were expressed as a percentage (g/100 g dry weight).

A 1 g tomato fruit sample, frozen in liquid nitrogen, was ground thoroughly with 5 mL of 80% ethanol to prepare a homogenate. The homogenate was mixed well and incubated in a water bath at 80–85 °C for 40 minutes. After cooling, 0.1 g of activated carbon was added for decolorization, and the mixture was stirred for 20 minutes. The sample was then centrifuged at 4000 rpm for 10 minutes. The supernatant was collected into a 25 mL volumetric flask (Sun J. et al., 2022; Sun L. et al., 2022). The extraction process was repeated for the remaining residue, and the resulting supernatants were combined. The final volume was adjusted to 25 mL with distilled water. The concentrations of sucrose, fructose, and glucose were then quantified using the anthrone colorimetric method (Sun L. et al., 2022).

Due to asynchronous fruit ripening, fruits were harvested sequentially. At each harvest, all mature fruits were counted, and each fruit was picked and immediately weighed using an electronic balance. Fruit number and individual fruit fresh weight were recorded for each harvest. At the end of the growth period, total yield per treatment was calculated as the sum of all recorded fruit fresh weights. Mean single fruit weight was calculated as the ratio of total yield to total fruit number for each treatment. Fruit dry weight was determined by oven-drying samples at 105 °C for 30 min, followed by drying at 70 °C to constant weight.

### 2.7 Enzyme activity assays

Enzyme extraction was performed based on the method described by Hussien and Doosh (2021), with minor modifications. For each treatment, 1 g of mature tomato pulp was homogenized in an ice bath with 10 mL of extraction buffer containing 0.1 mol/L phosphate buffer (pH 7.5), 10 mmol/L MgCl_2_, 1.0 mmol/L EDTA, 0.1% (v/v) Triton X-100, 0.1% β-mercaptoethanol, and 2% polyvinylpyrrolidone (PVP). The homogenate was transferred to a centrifuge tube and centrifuged at 10,000 rpm for 15 minutes at 4 °C. The resulting supernatant was collected and diluted fivefold for enzyme activity assays. The enzyme activity measurements were conducted following the protocol reported by Zhao (2021).

### 2.8 Statistical analyses

#### 2.8.1 Variance analysis

All data were analyzed using analysis of variance (ANOVA) followed by Duncan’s multiple range test in SPSS version 26.0 (IBM Corp., Armonk, NY, USA), with statistical significance set at *P* < 0.05. Prior to ANOVA, normality was assessed using the Shapiro–Wilk test and homogeneity of variances using Levene’s test. For a few cases showing deviations from normality or variance heterogeneity, log transformation was applied as a robustness check, and the statistical conclusions were consistent with those obtained from the original data.

#### 2.8.2 Interaction analysis

To investigate the interactive effects of relative air humidity (RH) and soil moisture (expressed as field capacity, FC) on physiological traits and sugar metabolism, two-way analysis of variance (two-way ANOVA) was performed to assess the main effects of RH and FC as well as their interaction (RH × FC).

#### 2.8.3 Simulation and prediction of tomato fruit sweetness

According to the sweetness calculation formula proposed by Wang (2002):


Sweetness value=fructose content×1.75 + glucose content×0.7 + sucrose content×1.00

Sweetness values were obtained under different air relative humidity and field capacity levels, and the optimal curve model was fitted.

#### 2.8.4 Gray correlation analysis

Gray correlation analysis was conducted to evaluate the impact of RH and RC on the tomato physiology, yield, and sugar content (Zheng J. et al., 2022). IBM SPSS Statistics 26 (SPSS Inc., Chicago, IL, USA) was used for the differences in the significance and the gray correlation analysis.

#### 2.8.5 Path analysis

Path analysis (Zelin et al., 2024) was used to explore the relationships between tomato sugar accumulation, physiological and biochemical indicators, and environmental factors. Prior to the path analysis, all variables were standardized.

## 3 Results

### 3.1 Net photosynthetic rate and related parameters

**Figure 1:**
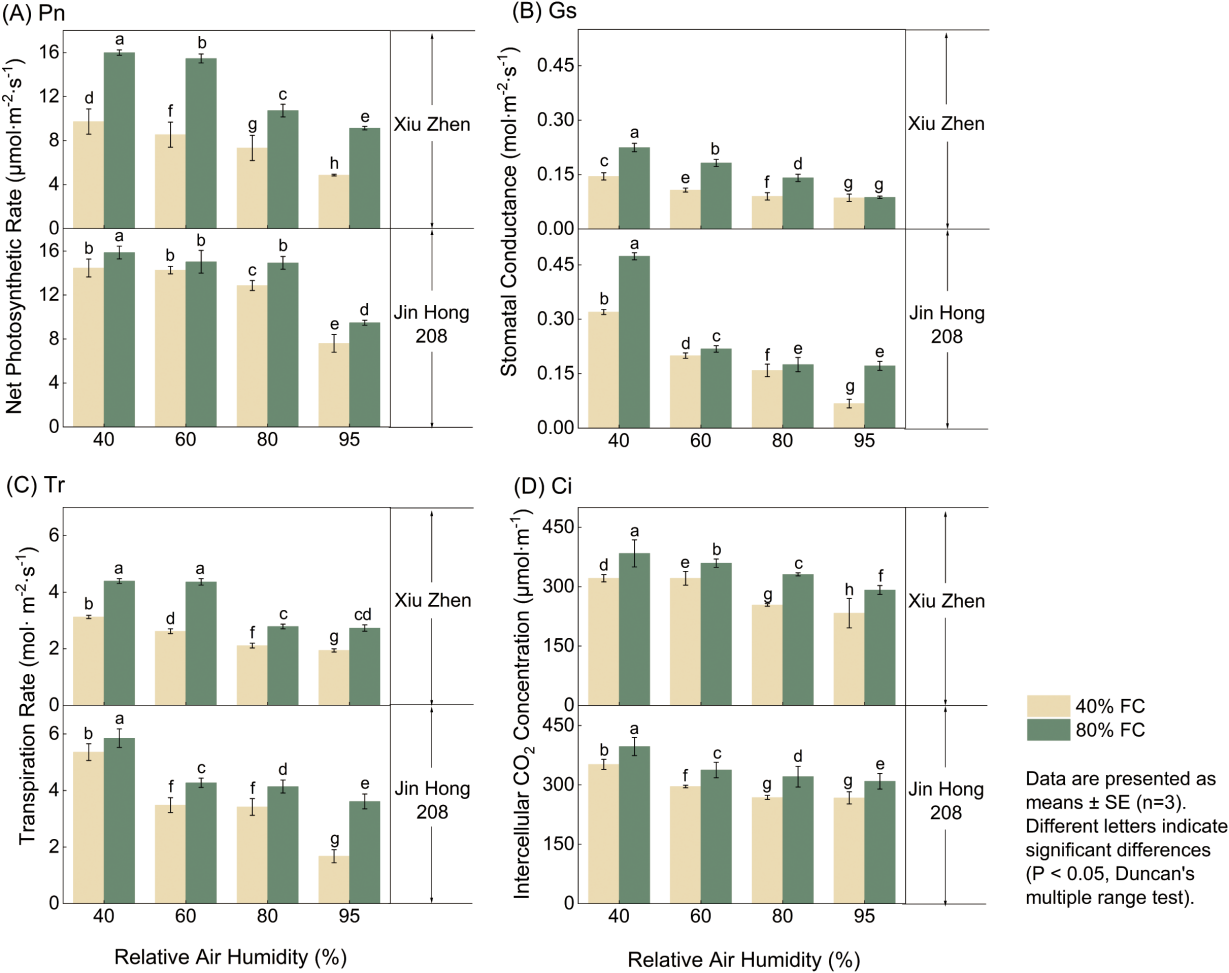
Effects of relative humidity (RH) and field capacity (FC) on leaf gas-exchange parameters—**(A)** net photosynthetic rate (Pn), **(B)** stomatal conductance (Gs), **(C)** transpiration rate (Tr), and **(D)** intercellular CO_2_ concentration (Ci) in two tomato cultivars (‘Xiuzhen’, ‘Jinhong 208’). Values are mean ± SE (n = 3). Different letters indicate significant differences among treatments (Duncan’s test, *P* < 0.05).

The net photosynthetic rate (Pn), stomatal conductance (Gs), transpiration rate (Tr), and intercellular CO_2_ concentration (Ci) of tomato cultivars ‘Xiuzhen’ and ‘Jinhong 208’ all decreased with increasing RH **Figure 1**). As shown in **Figures 1A**, under 40% soil moisture, when RH increased from 40% to 95%, the Pn of ‘Xiuzhen’ decreased from 9.73 to 4.86 μmol·m^−^²·s^−^¹, and that of ‘Jinhong 208’ dropped from 14.46 to 7.60 μmol·m^−^²·s^−^¹, indicating that high humidity significantly associated with lower photosynthesis. Increasing soil moisture enhanced Pn; for example, under 40% RH, the Pn of ‘Xiuzhen’ at 80% FC was 6.27 μmol·m^−^²·s^−^¹ higher than that at 40% FC, possibly due to improved water supply promoting stomatal opening. The change in stomatal conductance (Gs, **Figure 1B**) was consistent with that of Pn. Under the combination of 80% FC and 40% RH, ‘Xiuzhen’ reached the maximum Gs value of 0.2247 μmol·m^−^²·s^−^¹, while ‘Jinhong 208’ reached 0.4737 μmol·m^−^²·s^−^¹ under the same conditions—approximately 2.1 times that of ‘Xiuzhen’. The variation in transpiration rate (Tr, **Figure 1C**) showed a similar pattern, with the highest value (5.85 mol·m^−^²·s^−^¹) observed in ‘Jinhong 208’ under 80% FC and 40% RH treatment. For intercellular CO_2_ concentration (Ci, **Figure 1D**), at any given RH level, Ci under 80% FC was consistently higher than under 40% FC. For instance, under 40% RH, the Ci of ‘Xiuzhen’ at 80% FC was 62.90 μmol·m^−^²·s^−^¹ higher than at 40% FC, further confirming that higher soil moisture facilitates stomatal conductance.

### 3.2 Changes in chlorophyll content

**Figure 2:**
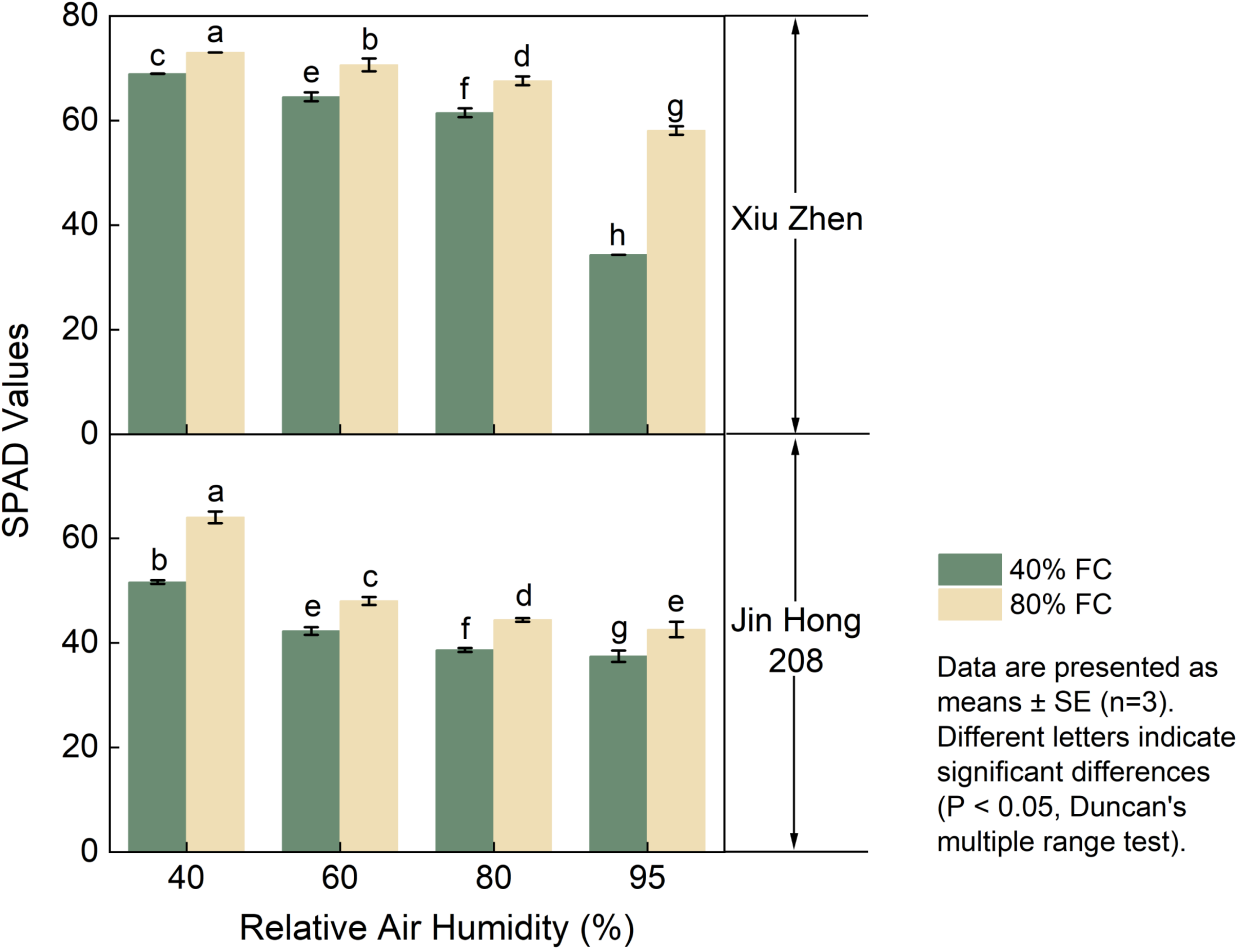
Activities of sugar-metabolizing enzymes in tomato fruits under RH×FC combinations: SPAD values. Values are mean ± SE (n = 3). Different letters indicate significant differences among treatments (Duncan’s test, *P* < 0.05).

SPAD was negatively correlated with RH and positively correlated with FC (**Figure 2**). Chlorophyll content declined with increasing RH. Under the combination of 80% FC and 40% RH, both cultivars reached their highest SPAD values, with 73.02 in ‘Xiuzhen’ and 64.00 in ‘Jinhong 208’. Higher soil moisture helped maintain relatively high SPAD values, but the extent of this enhancement weakened as RH increased.

### 3.3 Phloem flow rate, xylem flow rate and the transpiration rate of tomato fruit

**Figure 3:**
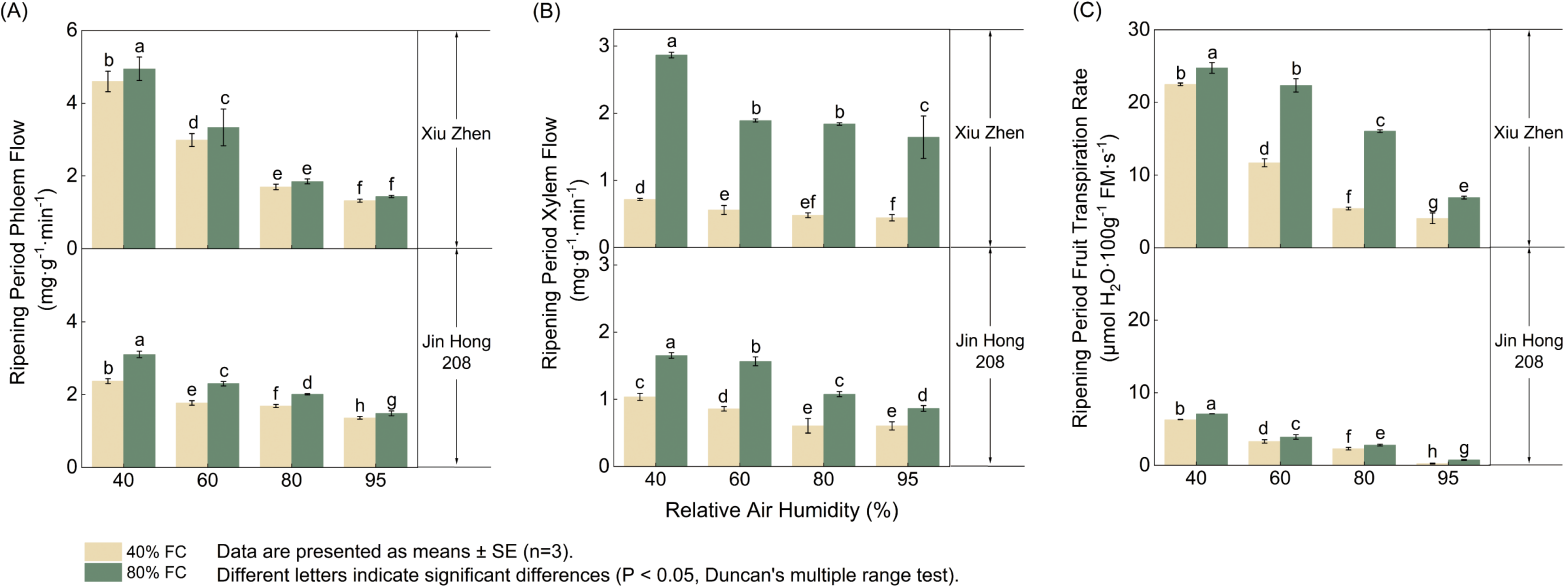
Effects of RH and FC on **(A)** phloem inflow, **(B)** xylem flow, and **(C)** fruit transpiration rate at the ripening stage in ‘Xiuzhen’ and ‘Jinhong 208’. Values are mean ± SE (n = 3); different letters denote significant differences (Duncan’s test, *P<* 0.05).

As shown in **Figure 3**, phloem flux, xylem flux, and fruit transpiration rate at the ripening stage all decreased with increasing RH but increased with higher FC. According to **Figures 3A**, under 40% FC, when RH increased from 40% to 95%, the phloem flux of ‘Xiuzhen’ declined from 4.60 to 1.32 mg·g^−^¹·min^−^¹, while that of ‘Jinhong 208’ decreased from 2.37 to 1.36 mg·g^−^¹·min^−^¹. Xylem flux was strongly affected by soil moisture. For ‘Xiuzhen’ at 40% RH, the xylem flux at 80% FC (2.87 mg·g^−^¹·min^−^¹) was 4.0 times higher than that at 40% FC (0.72 mg·g^−^¹·min^−^¹); for ‘Jinhong 208’, the increase was 1.6 times under the same conditions (**Figure 3B**). Changes in fruit transpiration rate (**Figure 3C**) were the most pronounced. For ‘Xiuzhen’, the highest value (24.75 μmol H_2_O·100 g^−^¹ FM·s^−^¹) occurred under the 40% RH and 80% FC combination, which was 6.1 times greater than the lowest value (4.05 μmol H_2_O·100 g^−^¹ FM·s^−^¹) observed under 95% RH and 40% FC. In contrast, the fruit transpiration rate of ‘Jinhong 208’ was lower than that of ‘Xiuzhen’ across all treatments, with its maximum value (7.10 μmol H_2_O·100 g^−^¹ FM·s^−^¹ under 40% RH and 80% FC) reaching only about 28.7% of that of ‘Xiuzhen’. These results clearly indicate that the combination of low air humidity and high soil moisture is most favorable for fruit water transport and transpiration.

### 3.4 Activity of sugar metabolizing enzymes in tomato fruit

**Figure 4:**
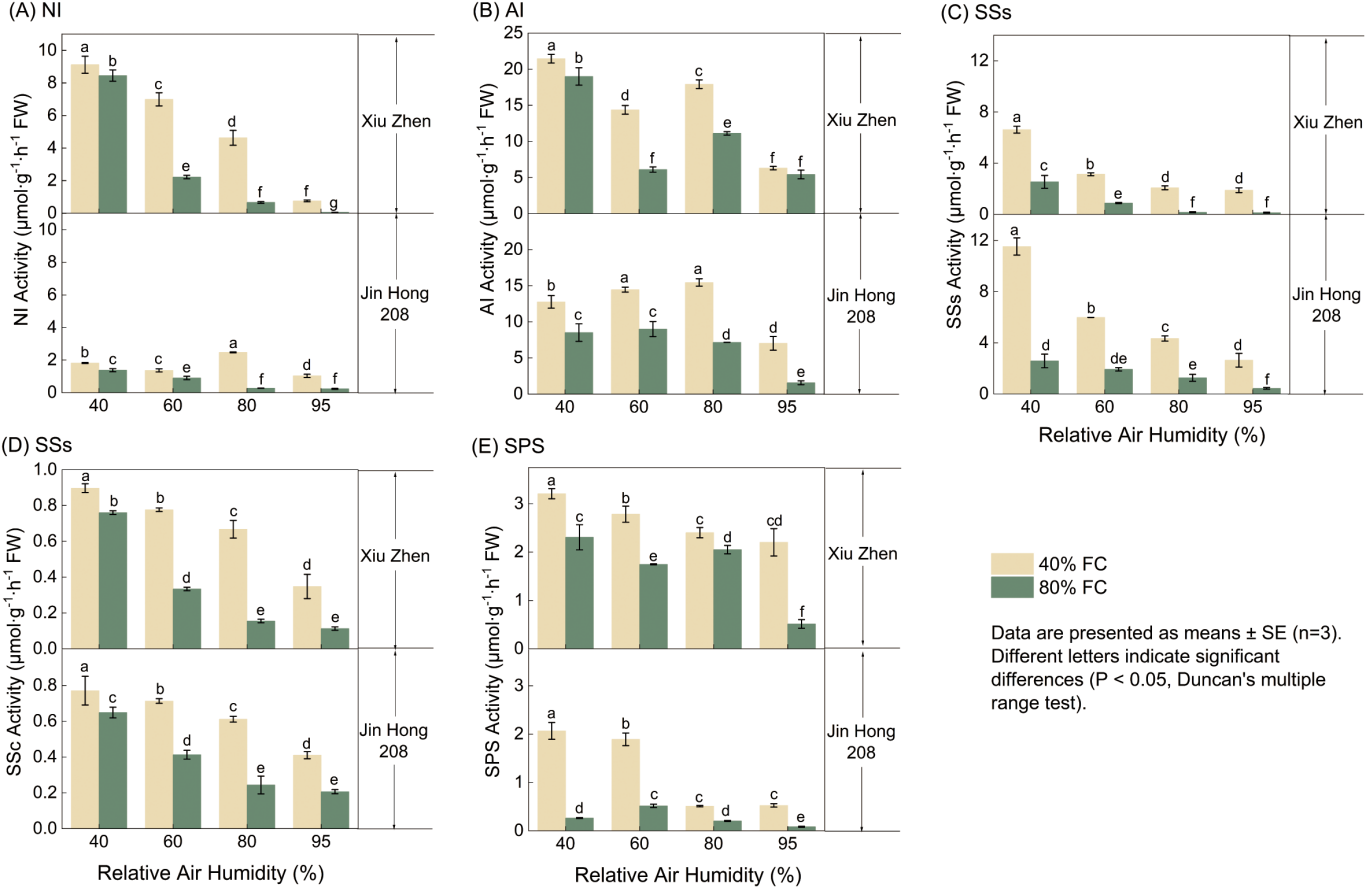
Activities of sugar-metabolizing enzymes in tomato fruits under RH×FC combinations: **(A)** neutral invertase (NI), **(B)** acid invertase (AI), **(C)** sucrose synthase—cleavage direction (SSs), **(D)** sucrose synthase—synthesis direction (SSc), and **(E)** sucrose-phosphate synthase (SPS). Values are mean ± SE (n = 3). Different letters indicate significant differences among treatments (Duncan’s test, *P* < 0.05).

The responses of key enzymes involved in tomato fruit sugar metabolism to RH and FC are shown in **Figure 4**. Overall, the activities of neutral invertase (NI), acid invertase (AI), sucrose synthase in the synthesis direction (SSs), sucrose synthase in the cleavage direction (SSc), and sucrose phosphate synthase (SPS) generally decreased with increasing RH and FC. In most cases, enzyme activities in ‘Xiuzhen’ were higher than those in ‘Jinhong 208’.

Detailed analysis showed that the activity of neutral invertase (NI, **Figure 4A**) was strongly influenced by environmental conditions. For ‘Xiuzhen’, the highest NI activity (9.12 μmol·g^−^¹·h^−^¹ FW) was observed under 40% RH and 40% FC, while under 95% RH and 80% FC, the activity sharply decreased to 0.05 μmol·g^−^¹·h^−^¹ FW—a reduction of 99.5%. For acid invertase (AI, **Figure 4B**), at 40% RH and 40% FC, the activity of ‘Xiuzhen’ (21.46 μmol·g^−^¹·h^−^¹ FW) was 8.70 μmol·g^−^¹·h^−^¹ FW higher than that of ‘Jinhong 208’ (12.76 μmol·g^−^¹·h^−^¹ FW). The activity of sucrose synthase in the synthesis direction (SSs, **Figure 4C**) showed a marked varietal difference. Under 40% RH and 40% FC, the SSs activity of ‘Jinhong 208’ (11.53 μmol·g^−^¹·h^−^¹ FW) was significantly higher than that of ‘Xiuzhen’ (6.62 μmol·g^−^¹·h^−^¹ FW), representing a 74.2% increase. However, when soil moisture increased to 80% FC, SSs activity in both cultivars declined sharply—dropping to 2.58 μmol·g^−^¹·h^−^¹ FW in ‘Jinhong 208’ and 2.54 μmol·g^−^¹·h^−^¹ FW in ‘Xiuzhen’. The activity of sucrose synthase in the cleavage direction (SSc, **Figure 4D**) fluctuated more markedly in ‘Xiuzhen’ across treatments. Its activity under 40% RH and 40% FC (0.90 μmol·g^−^¹·h^−^¹ FW) was 0.74 μmol·g^−^¹·h^−^¹ FW higher than under 80% RH and 80% FC (0.16 μmol·g^−^¹·h^−^¹ FW). In contrast, the SSc activity of ‘Jinhong 208’ varied less among treatments, with only a 0.56 μmol·g^−^¹·h^−^¹ FW difference between the highest and lowest values. For sucrose phosphate synthase (SPS, **Figure 4E**), the highest activity in ‘Xiuzhen’ (3.21 μmol·g^−^¹·h^−^¹ FW) occurred under 40% RH and 40% FC, and it decreased progressively with increasing RH, reaching the lowest value (0.51 μmol·g^−^¹·h^−^¹ FW) under 95% RH and 80% FC—an 84.0% reduction. The SPS activity of ‘Jinhong 208’ was generally lower, dropping to as low as 0.082 μmol·g^−^¹·h^−^¹ FW under 95% RH and 80% FC, significantly lower than in other treatments. These results indicate that the activities of sugar-metabolizing enzymes exhibit clear cultivar specificity and enzyme-type differences in response to water-related environmental conditions.

### 3.5 Sugar content, yield, and their correlations with humidity

**Figure 5:**
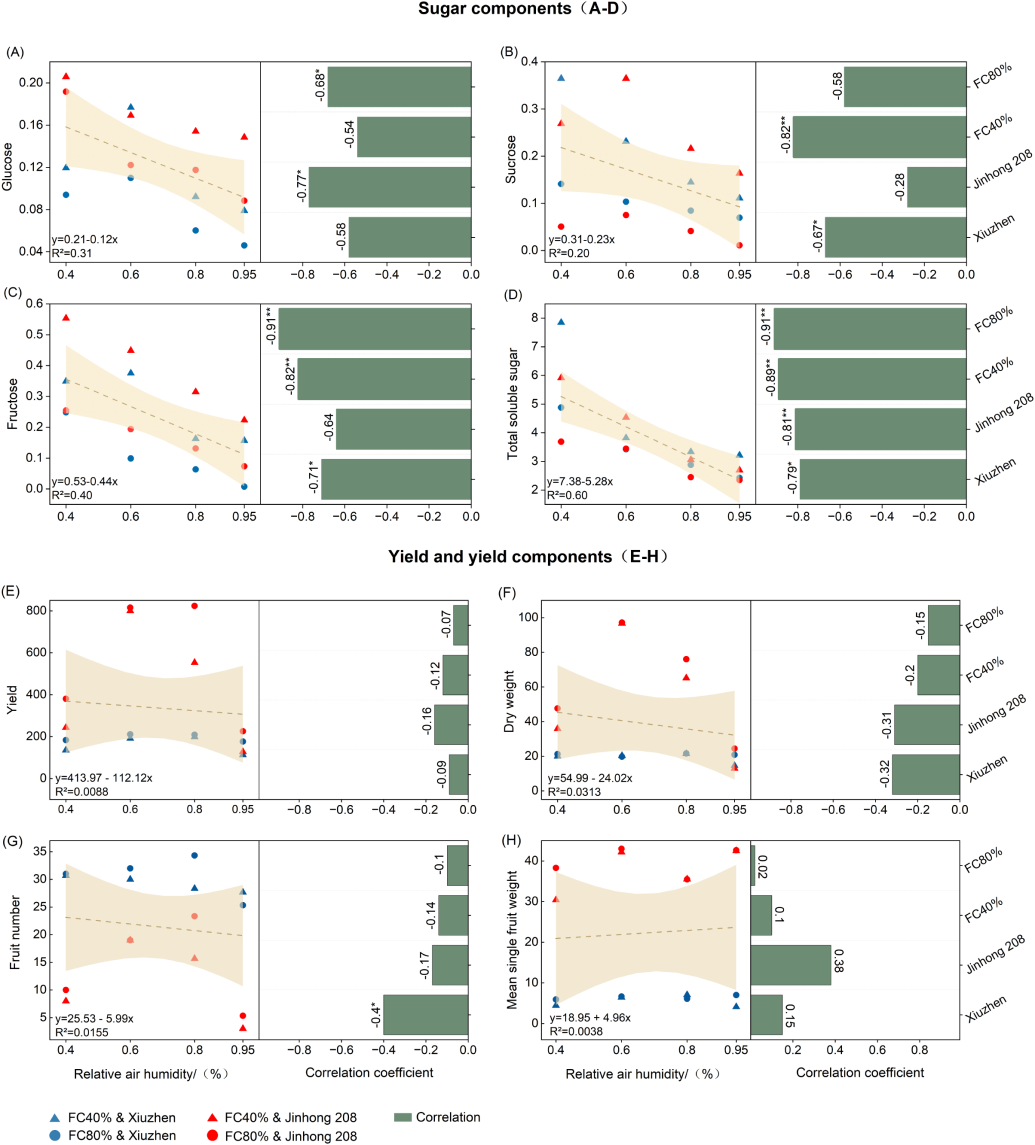
Effects of air humidity and field capacity on sugar components, yield, and yield-related traits of tomato. Relationships between relative humidity and **(A)** glucose, **(B)** sucrose, **(C)** fructose, **(D)** total soluble sugar content, **(E)** yield, **(F)** dry weight, **(G)** fruit number, and **(H)** mean single fruit weight are shown. Slopes and r values are shown in the panels; ** indicates *P* < 0.01, * indicates *P* < 0.05.

As shown in **Figure 5**, there is a significant negative correlation between fruit sugar accumulation and RH. Linear regression analysis indicates that the total soluble sugar content is most sensitive to changes in RH (slope = -5.28), followed by fructose (slope = -0.44), sucrose (slope = -0.23), and glucose (slope = -0.12). Comparisons between varieties show that the negative correlation of fructose (*r* = -0.71*) and sucrose (*r* = -0.67*) with RH is stronger in ‘Xiuzhen’ than in ‘Jinhong 208’, while the negative correlation of glucose (*r* = -0.77*) and total soluble sugars (*r* = -0.81**) with RH is more significant in ‘Jinhong 208’ than in ‘Xiuzhen’. This suggests that different varieties exhibit specificity in their response of sugar metabolism pathways to changes in humidity. Further analysis reveals that soil moisture associated with the relationship between humidity and sugar accumulation. Under the condition of FC 80%, the negative correlations of fructose and total soluble sugars with RH reached highly significant levels (both *r* = -0.91**), which were significantly stronger than under FC 40% treatment. In contrast, sucrose’s correlation with RH under FC 80% (*r* = -0.58) was weaker than under FC 40% conditions (*r* = -0.82**). This suggests that when soil moisture is sufficient, air humidity becomes the key factor limiting sugar accumulation, possibly due to the reduced transpiration and decreased phloem unloading efficiency under high humidity conditions.

In contrast to sugar traits, yield exhibited a much weaker response to changes in RH. Linear regression showed only a modest negative relationship between RH and total yield (y = 413.97 − 112.12x, *R*² = 0.0088), and correlation coefficients remained low in both cultivars (‘Xiuzhen’, *r* = −0.09; ‘Jinhong 208’, *r* = −0.16) and both FC levels (FC 40%, *r* = −0.12; FC 80%, *r* = −0.07). Yield components such as dry weight and fruit number also showed weak or cultivar-dependent associations with RH. Fruit number was significantly correlated with RH only in ‘Xiuzhen’ (*r* = −0.40*), suggesting varietal differences in yield sensitivity. Mean single fruit weight was essentially unaffected by RH (y = 18.95 + 4.96x, *R*² = 0.0038). Overall, these results indicate that the influence of humidity on yield formation is relatively limited compared with its pronounced effect on fruit sugar accumulation.

### 3.6 Effects of variety, RH, and FC on plant physiological processes and their interactions

The results of the analysis of variance for the three factors, variety, RH, and FC, are shown in **Table 1**. The results indicate that the main effects of variety, RH, and FC had highly significant effects on the majority of the measured parameters (*P* < 0.01), confirming that genetic background and moisture environment are key factors regulating tomato physiological metabolism. Interaction analysis revealed more complex regulatory relationships. The interaction between variety and RH (Variety × RH) had highly significant effects on all parameters except intercellular CO_2_ concentration (Ci), indicating that the two varieties exhibited widely different response patterns to changes in air humidity. The interaction between RH and FC (RH × FC) had highly significant effects on most indicators such as stomatal conductance (Gs) and transpiration rate (Tr), but had no significant effect on Pn (*P* = 0.151) and Ci (*P* = 0.447). Importantly, the three-factor interaction (Variety × RH × FC) had highly significant effects on multiple key parameters (*P* < 0.01), including net photosynthetic rate (Pn), stomatal conductance (Gs), chlorophyll content (SPAD values), xylem flow, and the activities of neutral invertase (NI) and acid invertase (AI). This indicates that the response of varieties to the moisture environment (RH and FC) is not independent, and the combined effect of high air humidity and different soil moisture levels significantly influences the physiological metabolism of the two varieties. However, the three-factor interaction did not have a significant effect on intercellular CO_2_ concentration (*P* = 0.814), fruit number (*P* = 0.221), mean single fruit weight (*P* = 0.105), and total soluble sugars (*P* = 0.225).

**Table 1:** Two- and three-way ANOVA for the effects of variety, relative humidity (RH), field capacity (FC), and their interactions on physiological, transport, and sugar-related variables in tomato.

Parameters	Variety	RH	FC	Variety×RH	Variety×FC	RH×FC	Variety×RH×FC
	P	P	P	P	P	P	P
Pn	**	**	**	**	**	0.151	**
Gs	**	**	**	**	**	**	**
Tr	**	**	**	**	0.246	**	**
Ci	0.223	**	**	*	0.201	0.447	0.814
SPAD values	**	**	**	**	**	**	**
Yield	**	**	**	**	**	**	**
Dry weight	**	**	**	**	**	**	*
Fruit number	**	**	**	**	0.181	**	0.221
Mean single fruit weight	**	**	*	**	0.314	*	0.105
Ripening period phloem flow	**	**	**	**	0.079	0.029	0.646
Ripening period xylem flow	**	**	**	**	**	**	**
Ripening period fruit transpiration rate	**	**	**	**	**	**	**
Neutral invertase	**	**	**	**	**	**	**
Acidic invertase	**	**	**	**	**	**	**
Sucrose Synthase-c	**	**	**	**	**	**	**
Sucrose Synthase-s	0.703	**	**	**	**	**	*
SPS	**	**	**	**	0.876	**	**
Fructose	**	**	**	**	**	**	**
Sucrose	**	**	**	**	**	**	**
Glucose	**	**	**	**	**	**	**
Soluble sugar	**	**	**	**	0.746	**	0.225

*: *P* < 0.05, **: *P* < 0.01.

### 3.7 Gray correlation between humidity, field capacity, and physiological/quality indices in tomato

**Figure 6:**
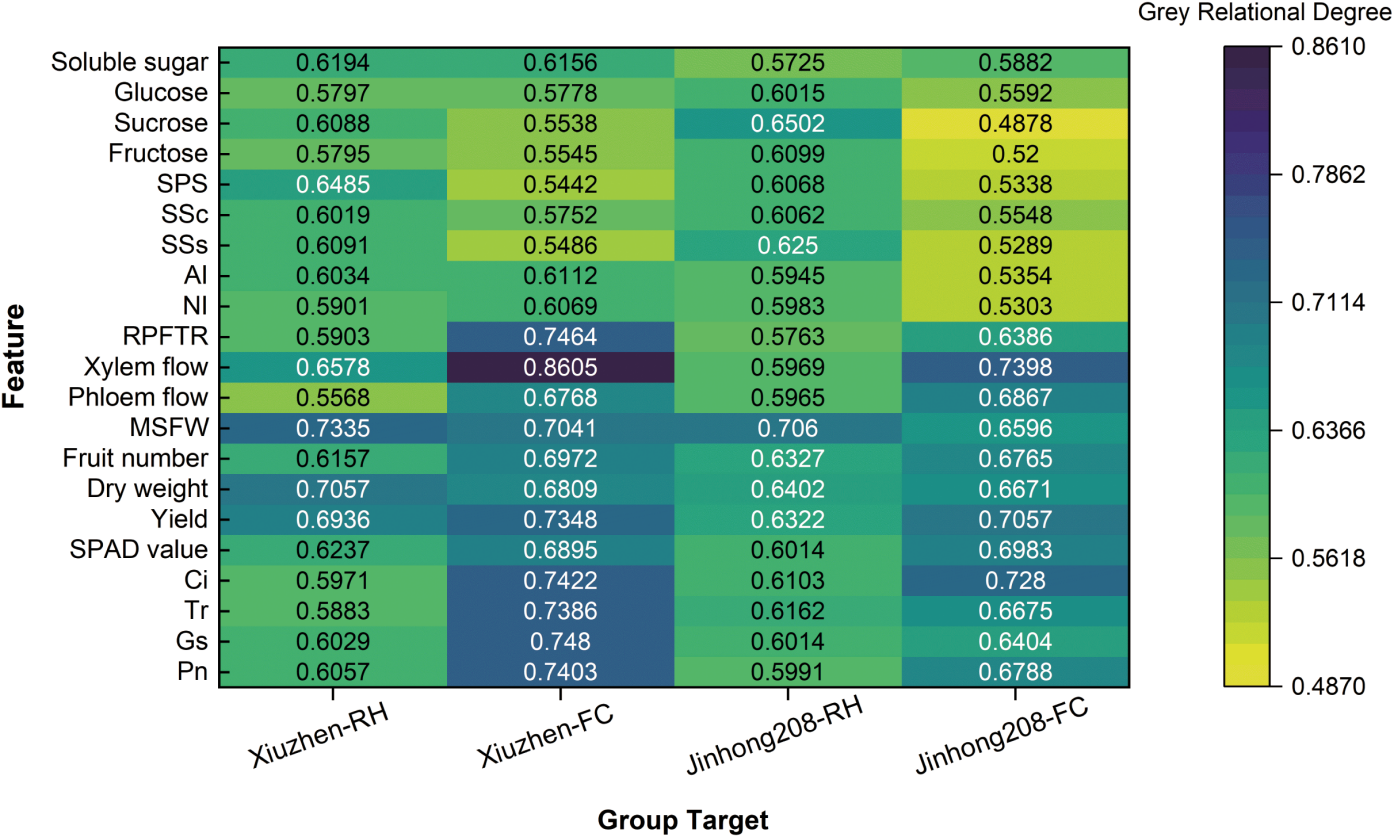
Grey relational analysis between moisture factors (RH, FC) and physiological/quality indices in ‘Xiuzhen’ and ‘Jinhong 208’. Bars show grey relational grades (ρ); higher values indicate stronger association. SPS, sucrose phosphate synthase; SSs, sucrose synthase (synthesis direction); SSc, sucrose synthase (cleavage direction); AI, acid invertase; NI, neutral invertase; MSFW, mean single fruit weight; RPFTR, ripening period fruit transpiration rate.

The results of the gray correlation analysis (**Figure 6**) reveal the degree of association between the physiological metabolic parameters of the two tomato varieties and RH and FC. Overall, the correlation between FC and the various parameters was higher than that with RH in most cases, and there were variety-specific response patterns. In the ‘Xiuzhen’ variety, the xylem flow had the highest correlation with FC (0.8605), indicating that its water conduction is significantly influenced by soil moisture. In addition, the correlations of net photosynthetic rate (Pn), stomatal conductance (Gs), and transpiration rate (Tr) with FC (0.7403, 0.7480, and 0.7386, respectively) were significantly higher than their correlations with RH (all below 0.61), suggesting a stronger association between the photosynthesis of this variety and soil moisture. In the ‘Jinhong 208’ variety, the correlation of sucrose content with RH was 0.6502, indicating that sucrose accumulation in this variety is more sensitive to air humidity. On the other hand, the xylem flow in ‘Jinhong 208’ also showed a relatively high correlation with FC (0.7398). Notably, the correlation of sugar metabolic enzymes (such as neutral invertase, acid invertase, and SPS) with RH was generally higher than their correlation with FC in both varieties, suggesting that air humidity may play a relatively dominant role in regulating the activity of sugar metabolic enzymes. Photosynthetic parameters (Pn, Gs, Tr, Ci) showed correlations with both water factors in the range of 0.58 to 0.75, indicating their joint influence by soil and atmospheric moisture.

### 3.8 Simulation and prediction of tomato fruit sweetness

**Figure 7:**
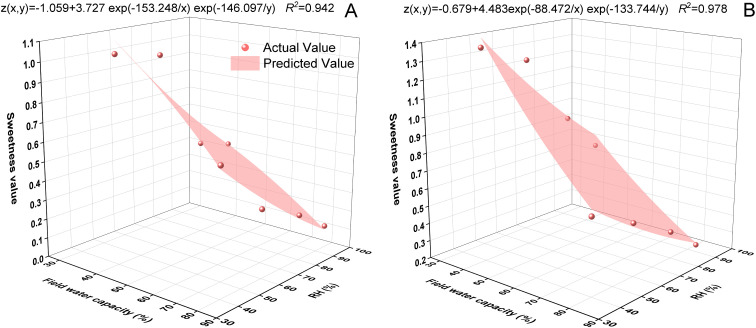
Response surfaces of predicted fruit sweetness as functions of FC and RH for **(A)** ‘Xiuzhen’ and **(B)** ‘Jinhong 208’ based on an exponential 2D model. Model fit (*R*²) is shown in each panel. Abbreviations: SPS, sucrose-phosphate synthase; SSs, sucrose synthase (cleavage direction); SSc, sucrose synthase (synthesis direction); AI, acid invertase; NI, neutral invertase; RPFTR, relative phloem flow to transpiration rate.

As shown in **Figure 7**, the exponential 2D model effectively reveals the relationship between fruit sweetness, FC, and RH. For the ‘Xiuzhen’ variety (**Figure 7A**), the coefficient of determination R2 = 0.942; in contrast, the model for the ‘Jinhong 208’ variety (**Figure 7B**) had a better fit, with R2 = 0.978, indicating excellent fitting accuracy. It can be observed that as FC and RH increase, the fruit sweetness gradually decreases. This indicates that moisture and humidity have a significant negative impact on fruit sweetness. Both varieties show the highest sweetness values at 40% FC and 40% RH, with ‘Jinhong 208’ having a higher sweetness value than ‘Xiuzhen’.

### 3.9 Path analysis

**Figure 8:**
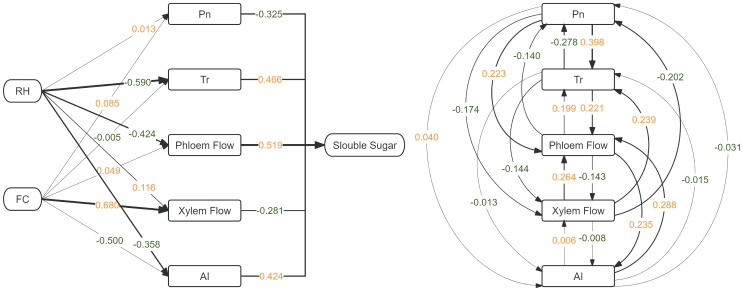
Path analysis of direct and indirect effects of RH and FC on soluble sugar content via net photosynthetic rate (Pn), transpiration (Tr), xylem flow, phloem flow, and acid invertase (AI). Numbers on arrows are standardized path coefficients; solid lines denote significant paths (*P* < 0.05). Positive and negative effects are indicated accordingly.

Based on the source–sink–transport theory, soluble sugar accumulation depends not only on carbon assimilation (source activity, Pn), but also on transport capacity (phloem flow, transpiration-driven mass flow) and sink metabolism (sugar metabolic enzyme activity). Therefore, these variables were incorporated into the path model to quantify the direct and indirect effects of RH and FC on sugar concentration. Acid invertase (AI) was chosen because it showed a relatively strong responsiveness to RH and FC in the gray relational analysis. The results of the path analysis (**Figure 8**) reveal potential direct and indirect pathways through which RH, FC, and fruit sugar content are affected by key physiological indicators (Pn, Tr, xylem, phloem, AI). The results indicate that different environmental factors influence sugar accumulation through different physiological pathways. For RH, its effect on sugar content is mainly mediated through indirect pathways. Among these, transpiration rate and phloem flow are key mediating factors, with direct path coefficients of –0.590 and –0.424, respectively, leading to a negative indirect effect of RH on sugar accumulation. The direct path coefficient for xylem flow is positive (path coefficient=0.116), but its positive effect is counteracted by negative effects transmitted through other factors. In contrast, FC exhibits a stronger direct effect on sugar content. The direct path coefficient for xylem flow is 0.680, which is negatively correlated with sugar accumulation, leading to a negative indirect effect of FC on sugar accumulation. Acid invertase (AI) shows a direct negative effect (–0.500), suggesting that high soil moisture may indirectly affect sugar metabolism via reduced AI activity. From the perspective of sugar content as the target variable, phloem flow (0.519) and AI (path coefficient 0.424) have significant positive direct effects on sugar accumulation, while net photosynthetic rate (–0.325) and xylem flow (–0.281) exhibit direct negative effects. This may be because, although high photosynthesis generates more sugar, the dilution effect of sugar caused by fruit cell water absorption and expansion under high moisture conditions leads to a decrease in sugar accumulation. This suggests that sugar content is primarily governed by the positive effects of phloem transport and AI activity, while complex feedback regulation triggered by photosynthetic product distribution and moisture conditions also plays a role. In this study, gray relational analysis and path analysis were applied to describe statistical associations among variables, and the coefficients represent relative association strength rather than causal physiological regulation.

## 4 Discussion

The interaction between soil moisture and air humidity on plant physiology and fruit quality has attracted increasing attention from researchers. Previous studies have mainly focused on the impact of individual factors on photosynthesis or sugar metabolism (Cui et al., 2020). Kawabata et al. (2005) reported that manipulating the ambient humidity to alter fruit transpiration resulted in a 40% decrease in tomato fruit growth rate, suggesting that that humidity-driven changes in fruit transpiration can affect water and dry matter accumulation. Liu et al. (2012) showed that water stress enhances sugar content by activating sugar metabolic enzymes, but did not fully resolve the coupling mechanism between photosynthesis and carbohydrate transport. Our results suggest that the RH effect on tomato sugar accumulation is mediated by coordinated changes in photosynthetic supply, vascular transport, and sugar-metabolic activity, and is strongly modulated by soil moisture availability. By combining the Exponential 2D model and fruit sweetness evaluation, this study also established a quantitative response-surface model to describe sweetness responses within the tested RH–FC range, providing a basis for hypothesis generation and future validation under commercial greenhouse conditions.

The path analysis model is consistent with the ‘source–sink–transport’ regulatory framework. RH mainly exerts an indirect negative effect on sugar accumulation by reducing VPD-driven transpiration and phloem transport, while FC promotes xylem flow but inhibits acid invertase activity. These results suggest that sugar accumulation is closely related to the coordinated balance between photosynthetic supply, vascular transport, and sugar-metabolism sinks. The positive pathways of phloem flow (0.519) and acid invertase (0.424) further support the important role of efficient transport and metabolic activity in the formation of fruit sweetness. Within this framework, the path model is used to organize statistically derived relationships among source activity, transport processes, and sink metabolism, and to explore plausible physiological linkages among these processes.

### 4.1 The effect of air humidity on fruit sugar content under conditions of sufficient soil moisture

Under conditions of sufficient soil moisture (FC 80%), fruit sugar content generally decreases as air humidity increases, consistent with previous reports (Chá et al., 2024; He et al., 2023). This phenomenon can be explained by several physiological processes. First, higher RH reduces the vapor pressure deficit and transpiration, which is often accompanied by reduced stomatal conductance and carbon assimilation, thereby limiting the source supply of photoassimilates (Falchi et al., 2020). Additionally, high RH significantly inhibits fruit transpiration, which further reduces the xylem water potential gradient and the driving force for phloem unloading. This phenomenon is consistent with the transpiration-phloem inflow counteracting effect observed in peach fruits by Morandi et al. (2007) and Kawabata et al. (2005). Furthermore, the decreases in NI and SPS activities at RH ≥ 80% suggest that high humidity may inhibit enzyme kinetics by lowering the osmotic potential in the cytoplasm. This interpretation is consistent with previous reports linking humidity to transpiration-driven transport, and our results further indicate that this limitation is most pronounced when soil water supply is non-limiting (FC 80%).

### 4.2 The effect of air humidity on fruit sugar content under conditions of insufficient soil moisture

When soil moisture is limited, the influence of RH on sugar accumulation is attenuated because carbon supply and transpiration are already constrained by root-zone water deficit (Fàbregas and Fernie, 2019). In the study by Fahad et al. (2017), they explored the growth coordination mechanisms of plants under soil moisture stress and pointed out that when plants face soil moisture shortage, they adjust leaf growth and transpiration to optimize the balance between water use and growth. Although increasing air humidity may slow down plant transpiration and lead to stomatal closure, under conditions of insufficient soil moisture, transpiration is already reduced. Therefore, the effect of air humidity on stomatal regulation is minimal. This is because, under water deficit conditions, water supply becomes the most critical factor affecting plant photosynthesis (Sun et al., 2020), and while changes in air humidity may have some effect on stomatal opening and closing, its impact on plant photosynthesis is far less significant than the direct shortage of water supply. Importantly, sugar reflects not only carbon assimilation but also fruit growth and water-related dilution; thus higher RH (lower VPD) can lower sugar via a dilution effect and altered partitioning, which may appear opposite to a purely photosynthesis-based expectation. This is consistent with previous studies under water-deficit conditions, and further suggests that soil moisture constrains the extent to which RH can regulate fruit sugar accumulation.

RH 40% significantly upregulated the activity of acid invertase (AI) and sucrose synthase (SS), suggesting that humidity stress may regulate sugar metabolism through osmotic stress signaling, possibly related to ABA accumulation (Durán-Soria et al., 2020; Waadt et al., 2022). These results are consistent with studies in lychee, where soil moisture stress enhanced the activity of AI, SS, and SPS, leading to increased sugar accumulation (Yang et al., 2013; Wang et al., 2019). Our study quantitatively characterized the coordinated role of air humidity in key sugar metabolism processes under water deficit conditions. The synchronized enhancement of sugar synthesis and metabolic conversion became the main driving force for sugar accumulation under low RH-FC conditions.

Under low soil moisture conditions, fruit sugar content is often higher than under high soil moisture conditions, even when air humidity is the same, for the following reasons: Although under low soil moisture conditions, plants close their stomata due to water scarcity, and photosynthetic efficiency decreases, the growth is suppressed, and the demand for carbohydrates for growth and dilution by fruit expansion are reduced, while soluble sugars can increase due to osmotic adjustment, leading to increased sugar accumulation, especially in fruits (Ma et al., 2022). That is, under low humidity conditions, there is a concentration effect on fruit growth (Chen et al., 2020). In contrast, under high soil moisture conditions, stomata remain open for a longer period, allowing the plant to perform photosynthesis more effectively. However, with faster photosynthesis and growth, sugars are primarily used to support growth, and due to the dilution effect, the sugar content in fruits is lower. Therefore, under low soil moisture conditions, plants may accumulate more sugar in the fruit by reducing growth and increasing sugar storage, whereas under high moisture conditions, more sugar is used for growth, leading to lower fruit sugar content (Bai et al., 2023).

### 4.3 The regulation of sugar accumulation by atmospheric relative humidity exhibits significant soil moisture dependence

The sensitivity of sugar accumulation to RH was strongly dependent on soil moisture availability. Under conditions of sufficient soil moisture (FC 80%), as RH increases, fruit sugar content decreases significantly. This is mainly due to the inhibition of transpiration, stomatal conductance, and photosynthetic rate in high-humidity environments, which weakens the synthesis of photosynthetic products and vascular transport efficiency (Zheng Y. et al., 2022). Both gray correlation and path analysis indicate that the negative effect of RH on sugar accumulation is primarily achieved via reduced photosynthesis, phloem transport, and the activity of sugar metabolic enzymes. In contrast, under conditions of insufficient soil moisture (FC 40%), the effect of air humidity on sugar accumulation is relatively weak. Low RH treatments can promote sugar accumulation by enhancing phloem flow and the activity of key enzymes (such as AI and SPS) (Luo et al., 2021). This suggests that when rhizosphere moisture is limited, tomato fruits compensate for insufficient photosynthetic supply by increasing transport and metabolic efficiency, thereby maintaining higher sugar content (Barbosa et al., 2023). These results are consistent with studies on crops such as lychee (Yang et al., 2013) and grapes (Ma et al., 2023; Morales et al., 2022), which collectively emphasize the central role of vascular transport in regulating sugar distribution. Overall, the effect of air humidity on sugar accumulation depends on soil moisture levels, with a stronger limiting effect under water-rich conditions and reduced sensitivity to RH changes under drought.

### 4.4 Analysis of varietal differences

The photosynthetic parameters (Pn, Gs) of ‘Xiuzhen’ tomato show higher gray correlation coefficients with relative humidity and field capacity, indicating greater sensitivity. This may be related to its stomatal regulation mechanism: for air humidity, the ‘Xiuzhen’ variety has a lower threshold for stomatal opening and closing, accelerating stomatal closure under high relative humidity to reduce excessive water loss, thus severely limiting photosynthetic carbon assimilation. In contrast, ‘Jinhong 208’ exhibits better photosynthetic stability, consistent with drought-tolerant traits reported previously (Conti et al., 2023). The phloem flow, xylem flow, and transpiration rate of ‘Xiuzhen’ decrease significantly with lower soil moisture and air humidity, indicating its response to stress by reducing water loss during water scarcity, which leads to a substantial increase in soluble sugar accumulation, in line with the plant’s ‘sugar priority’ strategy (Živanović et al., 2020). Compared to ‘Xiuzhen’, ‘Jinhong 208’ shows smaller changes in xylem flow and transpiration rate with variations in soil moisture and air humidity, demonstrating stronger water regulation ability. It is able to maintain stable water transport and transpiration under drought conditions, relying more on global water coordination (Sun et al., 2020). ‘Xiuzhen’ exhibits higher SPS activity under lower humidity and soil moisture conditions, suggesting it may optimize sugar accumulation by regulating the activity of key sugar metabolic enzymes. In contrast, ‘Jinhong 208’ shows relatively lower SPS and SSc activity, but higher AI activity, which gradually decreases with increasing humidity. This indicates that it maintains sugar supply by accelerating sucrose hydrolysis, supporting rapid fruit growth (Luo et al., 2021). These differentiated regulatory mechanisms collectively explain the differences in sugar accumulation patterns between the two tomato varieties.

This study was conducted under highly controlled conditions over a short period with only two cultivars. Thus, the RH–FC “optimal” combinations should be considered preliminary and limited to the tested range. Future studies should include a broader range of tomato genotypes and require multi-season, longer-term validation that also accounts for yield stability, disease risk, and economic costs.

## 5 Conclusion

Tomato sugar accumulation is jointly associated with RH and FC. High RH (≥80%) was associated with reduced photosynthesis and phloem transport, and coincided with lower sugar accumulation, whereas low RH was associated with higher transport and storage. In our experiment, humidity has a greater impact on sugar accumulation than on yield. Although higher FC (80%) improves photosynthetic activity, low FC (40%) was associated with higher sugar concentration despite reduced photosynthesis. FC shows a stronger direct association with sugar accumulation, while RH is mainly associated indirectly through transpiration and phloem flow. Within the tested conditions and experimental period, the highest sweetness was observed at 40% RH and 40% FC for both cultivars. Whether this combination is optimal under commercial greenhouse production requires longer-term validation across seasons and management regimes.

## Data Availability

The raw data supporting the conclusions of this article will be made available by the authors, without undue reservation.
